# Eliciting Nursing Students’ Preferred Designs for Pre-Class Preparation in Large-Group Teaching: An Action Research Study

**DOI:** 10.3390/nursrep16050176

**Published:** 2026-05-20

**Authors:** Anne Kristin Snibsøer, Christin Thompson, Venke Klubben Prytz

**Affiliations:** Department of Health and Caring Sciences, Faculty of Health and Social Sciences, Western Norway University of Applied Sciences, 5063 Bergen, Norway; chtho@hvl.no (C.T.); veklp@hvl.no (V.K.P.)

**Keywords:** nursing education, flipped classroom, pre-class preparation, action research, evidence-based practice

## Abstract

**Background:** The flipped classroom is an innovative student-centered teaching approach frequently applied in nursing education. The success of the approach relies on students coming prepared to class. Faculties play a critical role in facilitating students’ pre-class preparation. **Objective:** The objective of this study was to elicit nursing students’ preferred designs for pre-class preparation in large-group, flipped-classroom teaching in evidence-based practice, and to use these insights to inform practical, faculty-driven changes to course design and delivery. **Methods:** An action research study was conducted among bachelor’s students in nursing at a Norwegian university college. Data were collected through questionnaires with closed and open-ended questions, focus group interviews, and class meetings. Descriptive statistics and thematic analysis were applied to analyze data. Data were analyzed sequentially, and findings provided guidance for further actions. **Results:** The action was carried out and evaluated in two cohorts. The thematic analysis revealed one main theme—students need motivation for pre-class preparation—and three associated sub-themes: (1) Information: Communicate relevance and provide timely reminders, (2) Organization: Learning platform and workload, and (3) Engage learners: Diverse, interactive and aligned learning activities. **Conclusions:** Faculties can support motivation through clear communication of relevance, a well-organized learning platform, activating pre-class activities, and timely reminders. Successfully accommodating pre-class preparation for large-group teaching also appears to require coordinated faculty engagement and a shared commitment to student-centered approaches. Further evaluation is needed to determine which specific configurations work best in different contexts.

## 1. Introduction

To meet 21st-century challenges, a The Lancet report on “Education of Health Professionals for the 21st Century” [1] urged universities to move beyond memorization and transmission of facts to developing the competencies to access, appraise, analyze, and apply knowledge. In nursing education, there has been a shift away from transmissive models towards more student-centered teaching approaches [2]. Advancements in technology have enabled educators to adopt flexible, online and blended teaching approaches [3]. The flipped classroom is an innovative teaching strategy increasingly implemented in undergraduate nursing and health science education [4,5].

In a flipped classroom, students gain first exposure to new material before class so time in class time can be used to assimilate knowledge through problem-solving, discussions and collaborative learning [2,6]. The approach has the potential to enhance student engagement [2,7], stimulate reflection [8], promote deeper understanding [7], and cultivate 21st-century skills [9]. Systematic reviews have shown that a flipped classroom has an effect on student performance [10,11]. However, a prerequisite for the success of a flipped classroom is that students come prepared to class [11,12]. 

When designing pre-class preparation, instructors need to consider several factors. Students’ time to complete pre-class work is, according to Larson and Linell [12], a critical component. It includes time spent on pre-class work, adaptation to the flipped model, meeting deadlines, and students’ perceived workload. Moreover, the medium used to support students’ transition from passive to active learners should organize and structure content in a meaningful way [12].

Learning management systems serve as platforms for hosting and structuring educational information [7]. Video lectures, reading materials, quizzes, animations, and narrated power-points are commonly applied means to help students prepare [3,11,13]. High-quality flipped course materials and tools [14,15] and the quantity and difficulty of learning materials [16] may influence students’ preparation efforts. It is recommended to consider how assessment influences pre-class work and to ensure alignment between pre-class content and in-class activities [12]. Additionally, clear guidelines on use of pre-class time and course materials and timely support during out-of-class activities are important [11,12].

Although it remains unclear whether students are completing pre-class preparation before coming to class [12], several studies have described challenges regarding students’ pre-class preparation [7,11,12]. Frequently described challenges include increased time commitment and workload, lack of motivation, lack of guidance out of class, and poor quality of recorded lectures and videos [7,11]. The flipped classroom aligns with adult learning theory [2], in which adults are viewed as self-directed, problem-centered individuals who use internal motivation and experience to support lifelong learning [17]. Personal barriers to pre-class work may include difficulties with self-regulation, self-awareness, motivation, competing interests and metacognitive skills [12].

Norwegian white papers [18] emphasize the need to strengthen student-active teaching approaches. At the same time, higher education faces increasing economic pressure [19], resulting in reductions in teaching hours. In a nursing course at a Norwegian university college, a flipped-classroom model was implemented with varying success. The faculty experienced that most students tended to come prepared for small-group activities, but not for large-group teachings.

Previous systematic reviews on the flipped classroom in nursing education have explored academic performance [2], satisfaction and self-efficacy [5], engagement [20], pedagogical design [13], self-directed learning [21], effectiveness [22,23,24], and course outcomes and learning skills [25]. All reviews included content on pre-class activities, except Tan et al. [24]. However, none explicitly described how pre-class preparation should be structured for large-group teaching, nor how students themselves would design pre-class activities that are feasible and motivating for large-group teaching formats.

Inspired by the scholarship of teaching and learning (SoTL) [26], we adopted an exploratory, systematic approach to examine our current practice in preparation for large-group teachings. The objective of this study was to elicit nursing students’ preferred designs for pre-class preparation in large-group, flipped-classroom teaching in evidence-based practice, and to use these insights to inform practical, faculty-driven changes to course design and delivery.

## 2. Methods

### 2.1. Design

We applied McNiff’s [27] model of action research as our framework. Action research is a systematic, reflective inquiry process in which practitioners investigate their own practice with the aim of improving it [27]. We found the design appropriate because we intended to challenge and improve our teaching practice.

**Figure 1:**
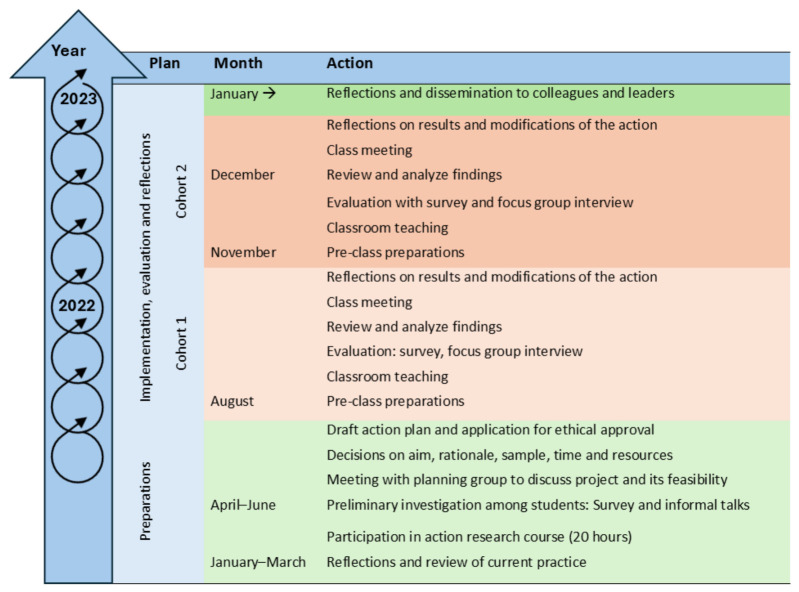
Timeframe of action research project.

The study proceeded as a cyclical process in five sequential phases: (1) identify a problem, (2) plan an action, (3) test the action, (4) evaluate, and (5) modify practice (Figure 1). We began by identifying a problem through reflection and preliminary investigations. We observed that students often came unprepared to large-group teaching sessions, despite instructions to complete pre-class readings. In our reflections, we asked how we could better elicit students’ pre-class preparation. We planned an action and tested it with a cohort of nursing students, evaluated it and reflected upon the results. We modified practice based on the evaluation, and the refined action was subsequently tested in a new cohort of nursing students. Data were collected and analyzed continuously, and the results from each cycle guided subsequent actions. For reporting the study, we applied “Best practices in the reporting of participatory action research” [28].

### 2.2. Setting and Participants

The setting of this study was flipped-classroom teaching in evidence-based practice (EBP) that took place in a three-year bachelor’s program in nursing at a Norwegian university college. The teaching spanned ten days and comprised pre-class, in-class and post-class activities, following a common flipped-classroom model [22]. Pre-class materials were provided on the students’ learning management system, Canvas [29], and included videos, readings, and reflection assignments. In-class teaching was scheduled over three days (2 + 6 + 1 h), and included brief introductory lectures, small-group tasks and discussions organized according to the IGP (individual, group, plenary) principle, Kahoot quizzes, and small-group work (1 h) with plenary feedback. Attendance at these large-group sessions was voluntary. The post-class activity was a mandatory written assignment (pass/fail), in which students selected one of five health claims and evaluated the advice by comparing it with evidence from synthesized summaries.

Participants were second-year bachelor’s students in nursing (*n* = 175). The semester comprised eight weeks of teaching and ten weeks of clinical placement studies. The students were divided into two cohorts (C1: *n* = 75 and C2: *n* = 100), and all teaching sessions were performed twice. The teaching in EBP was scheduled in August (C1) and November 2022 (C2).

Information about the study was posted on the students’ learning platform before the teaching began. All second-year students in the respective cohorts were invited to participate. We recruited students who attended the in-class teaching on days 1 and 2. For the survey, one reminder was sent.

### 2.3. The Action

The action was developed based on recommendations for pre-class preparation described in systematic reviews available at the time of the study [2,11,13], feedback provided by previous students, and faculty experience. It included three components: (1) information about the flipped classroom, (2) a dedicated Canvas page with pre-class learning activities, and (3) allocated time for self-study.

Information about the concept of the flipped classroom and the importance of pre-class preparation was communicated orally at the start of the semester and in writing on Canvas. During the oral briefing, the Canvas page containing the EBP learning activities was specifically highlighted.

**Figure 2:**
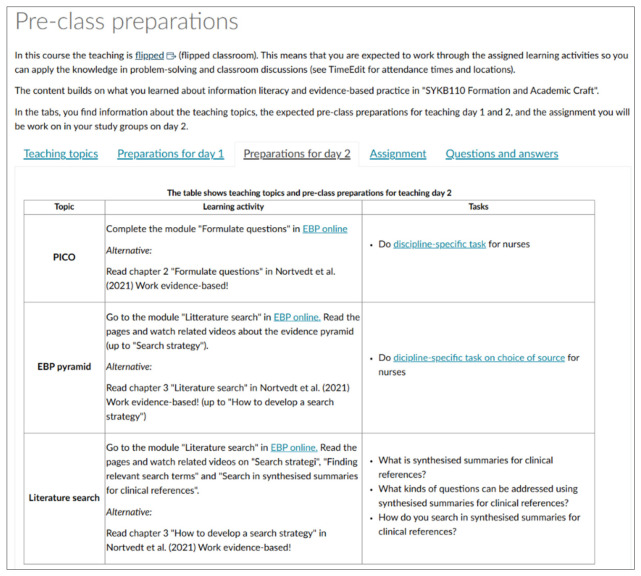
Screenshot of Canvas page. Learning materials were available from the digital learning resource “EBP online” [30] and the course textbook of Nortvedt et al. [31].

All pre-class learning activities were presented on a single page in Canvas (https://www.hvl.no/en/hvl-students/learning-management-system/ access date on 8 May 2026), in tabs for each teaching day (Figure 2). Students could choose between materials in a specified digital learning resource [30] or readings from the course textbook [31]. They could also choose to complete exercises, quizzes and reflection tasks.

The digital learning materials we applied were collated in the Canvas resource “EBP online” (KBP på tvers) [30]. This educational resource is developed by experts in EBP and contains high-quality videos and interactive learning materials. The resource has restricted access, but all Norwegian healthcare education programs can request entry. We selected content that aligned with our teaching sessions. In total, we recommended 23 pages that included 14 videos (ranging from 2:37 to 8:04 min), one podcast, and ten interactive learning resources such as drag-and-drop tasks, fill-in-the-word exercises, and dialogue cards. Additionally, we provided links to five specific pages from “Behind the Headlines” [32].

Times for pre-class activities were specified in the students’ schedule TimeEdit [33]. Pre-class activities for the second day were estimated to require three hours. To clarify our expectations, we scheduled two hours of self-study for the first day and five hours for the second day of classroom teaching.

### 2.4. Measures

Data were collected via a study-specific questionnaire (closed and open-ended items), focus group interviews, and class meetings. The questionnaire was developed for this study and was not tested for validity nor reliability prior to use. It addressed how students prepared for teaching and demographic variables. Close-ended items asked whether students usually prepared for large- and small-group teaching sessions and whether they had prepared for the day’s session. Two open-ended questions invited students to describe how they had prepared for the day’s teaching session or, if they had not prepared, to explain why. An additional open-ended item asked students to suggest ways of communicating pre-class preparation that would engage them and improve preparedness for themselves and their peers.

We pilot-tested the questionnaire during preliminary investigations and made minor adjustments to wording and response options. We added “sometimes” as an additional response for the preparedness questions, and changed the items about how they had prepared/why not from close-ended to open-ended formats. The survey was administered on the first day of teaching.

The focus group interviews were conducted on the second day of teaching. We used a semi-structured interview guide. Students were asked how they preferred pre-class preparation to be organized to enhance preparedness for themselves and their peers. The main question was “*If you had unlimited resources available, how would you design the pre-class preparation for this teaching in evidence-based practice for yourself and your fellow students?*” Follow up questions explored what in the proposed preparation had worked well and what had not worked. Each interview lasted up to 30 min. All data were collected by lecturers not involved in teaching.

The official class meetings were held once for each cohort during their eight-week teaching period. These meetings covered topics beyond pre-class preparation and EBP teaching. We also met with students on other occasions to discuss preparation and teaching informally. In those meetings, we asked students for additional input and further elaboration on the findings from the survey and focus group interviews. Brief field notes were taken from these meetings.

### 2.5. Analysis

Descriptive analyses were used to summarize demographic characteristics and preparedness for class. We assessed distributional differences between cohorts for categorial variables using exact methods appropriate for small cell counts. Fisher’s Exact Test was used to compare the two cohorts on gender (woman, male), prior studies (yes, no), and preparedness for the day’s teaching (yes, no). The Fisher–Freeman–Halton Exact Test was used to assess differences in preparedness for large-group and small-group teaching (each: yes, sometimes, no). Differences in age were assessed with the Mann–Whitney U Test. The open-ended questions about how students had prepared for the day’s teaching and reasons for not preparing were organized into themes, categorized and summarized. *p*-values less than 0.05 indicated statistical significance. The analyses were performed in the statistical software IBM SPSS Statistics version 31.0.1.0 [34].

Qualitative data were analyzed using thematic analysis, inspired by Braun and Clarke’s [35] six phases: (1) familiarizing with data, (2) generating initial codes, (3) searching for themes, (4) reviewing themes, (5) defining and naming themes, and (6) producing a report. We used an inductive approach since we were interested in students’ perspectives. Two authors (C.T., V.K.P.) began familiarization by transcribing the interviews verbatim and reading the data several times to identify meaning and patterns. They independently generate initial codes and started to search for themes and sub-themes. Thereafter, the authors held several meetings to further discuss and categorize codes, identify patterns and explore how codes could be combined into broader themes. Themes were refined through iterative discussion and reflection among all authors. All authors participated in naming and reviewing themes, discarding those not relevant to the research question, and selecting representative quotations. Aware that our roles as educators could influence interpretation, we adopted a reflexive approach throughout the analysis. Disagreements in the analysis process were solved by discussion and consensus in regular meetings. We did not apply consensus coding or inter-coder agreement, as this does not align with the analytic process of reflexive thematic analysis [35]. All authors engaged in the reflexive discussion and in finalizing the analytic text. Coding was performed manually, and the analytic process was reflected by notes in a working document. No qualitative data analysis software was used. Table 1 provides an example of excerpts and sub-themes constituting the theme.

To strengthen trustworthiness, we triangulated data sources, by cross-validating the findings from the questionnaire and focus group interviews. Data from class meetings were used as supplementary information, to further understand and explain survey and interview findings.

### 2.6. Project Group

The project group consisted of two lecturers and one associate professor. The two lecturers were fairly new to the university college. They were trained nurses with pedagogical education and had prior experience with small-group teaching. The associate professor had extensive experience in teaching EBP at both the bachelor’s and master’s levels. Her pre-understanding was that many bachelor students found EBP challenging to comprehend and that few came prepared for large-group teaching. All members were involved in developing the action plan. To differentiate between teaching and evaluation, the associate professor conducted the classroom teaching, while the lecturers were responsible for data collection and preliminary data analysis.

### 2.7. Ethical Considerations

The Norwegian Agency for Shared Services in Education and Research (Sikt) (ref. 933252) approved the study. Participation was voluntary, and consent was given by completing the anonymous questionnaire and giving consent prior to participation in the focus group interviews. All personal data has been treated confidentially and in accordance with privacy regulations.

## 3. Evaluation and Reflections

### 3.1. Evaluation Cohort 1

The evaluation of the first action drew on data from the survey, one focus group interview, and class meetings. Of the 75 eligible students, 41 attended the teaching session and 27 completed the questionnaire, yielding an overall response rate of 36%. The respondents were predominantly women (85%), with a mean age of 22.7 (SD ± 4.3) years (range 19–38 years) (Table 2).

The results indicated that 62% (*n* = 16) had prepared for the classroom teaching in EBP, mainly by using resources provided in Canvas (Table 3).

The results from the focus group interview and open-ended questions indicated that the students were satisfied with the structure and organization of the Canvas page. They pointed out the importance of a clear layout, easily accessible information and preparation materials, and a manageable workload. Several students said they would appreciate a reminder and suggested an announcement posted two days before class with a brief explanation of why they should prepare. One student said, “*Some teachers are good at posting an announcement one to two days before, about what we should read before the teaching session. Then I’m a bit more on [engaged], because it gives me a reminder”* (Interview 1.1). In the class meeting, it emerged that it was easier to be motivated when they were explicitly told that they needed to prepare, rather than having to read this information on their own.

### 3.2. Reflections Cohort 1 and Further Action

Due to low response rate, we discussed whether to continue the action process. We observed limited engagement for pre-class preparation and for participation in the study. Further, the results from the survey and focus group interview did not provide substantial information to act on. However, because the students were at the beginning of their second year and still orienting themselves in a new course, we decided to continue the action with the next cohort of students, incorporating incremental adjustments into the action.

For the second cohort, we kept the same Canvas setup and the scheduled timeframe in TimeEdit. The only change we made was to add an oral announcement (voice message) in Canvas two days before the teaching session, explaining why and how the students should prepare. We also shared information from the first cohort’s experiences with the pre-class preparation.

### 3.3. Evaluation Cohort 2

The evaluation of the second cohort was based on findings from the survey, five focus group interviews, and class meetings. Of the 100 eligible students, 60 attended the teaching session and 54 completed the questionnaire, yielding an overall response rate of 54%. The respondents were predominantly women (89%), with a mean age of 22.3 (SD ± 3.1) years (range 19–34 years) (Table 2).

The results indicated that 76% (*n* = 41) had prepared for the classroom teaching, mainly by using resources available on Canvas (*n* = 35) (Table 3).

The thematic analysis revealed one main theme—students need motivation for pre-class preparation—and three associated sub-themes: (1) Information: Communicate relevance and provide timely reminders, (2) Organization: Learning platform and workload, and (3) Engage learners: Diverse, interactive and aligned learning activities.

### 3.4. Information: Communicate Relevance and Provide Timely Reminders

Several students reported that explicit communication about why pre-class preparation matters, together with short reminders a few days before class, increased their motivation to prepare. Students said that they needed to understand why they should prepare and what relevance the preparation had for teaching, exams and further professional work life. One student explained, *“…I think they should say why you have to prepare. Like because we are going to have a discussion or do some exercises”* (Interview 2.3). Another commented, *“…maybe a nurse who could say something very specific about how they use it in their daily work, doctors … That is, people who could tell you why this is relevant for you to learn”* (Interview 2.5). A third noted that when many assignments competed for attention, non-mandatory preparation felt optional unless their relevance was made explicit: “*You might be a little pressed, so you’d rather work on that instead of the preparation to class that aren’t mandatory… you have to make it a little more attractive to us actually want to put in that kind of work before the lecture”* (Interview 2.3).

Several students also reported that reminders, preferably from teachers in class or via the learning platform a few days beforehand, helped them complete preparatory work. As one student described, “*I think the voice message we received from the lecturer was motivating, as it gave me an extra push and motivation to do some reading. Then she could have mentioned a little more about why this is an exciting and important topic to dive into, as that can be a motivating factor. Nevertheless, I think it was a little extra push to hear the teacher say it in her own words, why it was important to prepare, instead of having to read a long and dry text”* (Survey 2).

### 3.5. Organization: Learning Platform and Workload

Students acknowledged that a clearly organized learning platform, where information and learning resources were easy to find, increased motivation for pre-class activities. At the same time, many found the volume of activities overwhelming, especially as the topics were new. For example, one student appreciated a “*recipe or a template on Canvas that you could follow to the letter”* (Interview 2.3), while another described becoming overwhelmed by the information: “*there were so many things that I completely lost track, and it was like… I’m never going to manage this, you know…”* (Interview 2.5).

Students experienced the eight-week teaching period as demanding, with six mandatory small-group sessions, a written assignment, and an approaching exam. Several students claimed they needed help with prioritization. Suggestions included spreading the teaching and pre-class preparation across a longer period and signposting essential versus optional materials. To make the workload more manageable one student proposed to mark the materials with statements like *“this you need to know and this you can read more about*” (Interview 2.4). One student commented, *“I feel like I have so many things going on that I can’t put my soul into one task before I have to start on the next one”* (Interview 2.1).

### 3.6. Engage Learners: Diverse, Interactive and Aligned Learning Activities

Students welcomed pre-class preparation that was composed of varied learning activities that were activating and not too extensive. They wanted additional approaches to readings and videos, and mentioned quizzes and assignments where they could use their knowledge as examples of alternative approaches. *“I think that there were several videos that were perhaps a bit repetitive and perhaps unnecessary. But the tasks that came afterwards, I kind of learned more from them”* (Interview 2.3).

Many students commented on previous and actual teaching, and stressed the importance of alignment between pre-class preparation and in-class activities. Several commented that lecturers sometimes duplicate the preparatory material, making one of the two seem redundant. One said, “*I felt, at least, that the first lecture was a mere repetition of the preparation we had done. So then I really felt that I could only have chosen one of the parts…”* (Interview 2.2). In contrast, when in-class activities extended pre-class work, such as group assignments that prompted discussion and teacher facilitation, students found the combination effective: *“When I sat at home yesterday and prepared, I understood pretty well. Then I came here today and got it a little doubled up and that, for me, it works fine… And she had group assignments where we were supposed to discuss among ourselves and then she talked about it out loud. And that, for me, it works perfectly the way it was today actually”* (Interview 2.1).

Students preferred small-groups teaching and group assignments facilitated by teachers where they could ask questions in a safe environment. Several students experienced barriers to ask questions in a large classroom and in the discussion forum on the learning platform. Small groups fostered responsibility, psychological safety for asking questions, and greater accountability to peers, all of which increased motivation to prepare. As one student put it, “*If you do not prepare for small-group teaching, it is worse than if you do not prepare for an auditorium lecture (large-group teaching)… You have a greater sense of responsibility, maybe because it affects others … And it is easier to feel seen in a smaller group”* (Interview 2.2).

### 3.7. Reflections Cohort 2 and Further Action

Based on the findings from cohort 2, we recognized that successful engagement in pre-class preparation depended on more than the actions of a single course instructor. Effective preparation requires collective pedagogical commitments across the faculty and coordination of student-centered teaching approaches.

Due to changes in curriculum, we could not continue the planned action research cycles. However, we did implement some further actions. First, heightened awareness of the importance of preparatory information, workload balance, time, and in-class pedagogical approaches led us to make more conscious choices when planning and designing pre-class preparation. Second, recognizing that students’ readiness is shaped by prior teaching practices, we have discussed the study findings and reached a consensus within the faculty on how, when and where pre-class activities should be used in large-group teachings. By doing this we aim to reduce concurrent demands, clarify expectations for preparedness, and distribute workload more evenly. Finally, by systematically reflecting on our teaching practice and acknowledging the insight we gained, we have disseminated the study at conferences and staff meetings and encouraged colleges to engage in the scholarship of teaching and learning.

## 4. Discussion

This study confirms that students need motivation to complete pre-class preparation and that faculties can support this by providing information that communicates relevance, organize learning platforms and workload to limit overload, and engage learners with intentional pedagogical approaches. We also found that accommodating pre-class preparation to large-group teachings requires engagement from a collaborative faculty committed to student-centered teaching approaches.

Our findings suggested that making the relevance of pre-class activities more explicit may increase students’ motivation to prepare. A recent systematic review offering recommendations for pre-class work [36] does not explicitly mention relevance, but it does emphasize that faculties should communicate the purpose of the flipped classroom and the link between pre- and in-class activities. Interestingly, we believed we had supplied students with relevant information. Since some students may perceive the flipped model as requiring more time and work than a traditionally structured course [11], we communicated relevance of pre-class work primarily in relation to upcoming learning and teaching activities. Our information briefly described the flipped-classroom concept and highlighted potential benefits of pre-class activities for in-class participation and post-class work, but did not explicitly connect preparatory activities to future professional practice. Given adult learners’ need to understand why they should learn something [17], it is possible that more extended information explicitly tying preparation to later work would be beneficial. Further examination is needed to determine whether such changes would actually increase preparation or motivation.

Students across both cohorts proposed reminders to encourage engagement with pre-class activities. From implementation science we know that reminders can have significant effects on clinical practice outcome [37]. Still, for the first cohort we found reminders unnecessary because we considered students to be self-driven based on the oral and written information we provided two weeks before teaching. For the second cohort, however, we built on our findings and supplied students with a voice message reminder. Although survey findings showed that the majority of students in both cohorts came prepared to class, our observations indicated that students in the second cohort had put more effort into their preparation and used more of the recommended learning resources. It is possible that the voice reminder and information shared from the first cohort influenced the second cohort. However, other plausible explanations include inter-student communication across cohorts, low first-attempt pass rates on the assignment, and also timing. Thus, further investigations are needed to explore the relative impact of these influences.

We invested time into designing an organized learning platform and students generally described it as structured, with information and learning resources easily accessible. Previous research has indicated that easy access to course material can increase motivation [38]. At the same time, students reported feeling overwhelmed by the volume of information, a pattern that was especially evident in the second cohort and reflected in survey results, interviews and class meetings. One possible explanation is that this cohort was closer to the upcoming exam and felt short of time. Another possibility may be that we attempted to accommodate diverse learning preferences by providing both video resources and course readings, together with exercises, quizzes and reflection tasks, which may have increased perceived workload. With reductions in teaching hours and increased expectations for self-study, we outlined several hours of pre-class activities. Our planning assumed self-driven students. However, we may have overestimated students’ intrinsic motivation for pre-class work and underestimated the time needed for completion. There is no consensus on an appropriate duration for pre-class work [12]. It is possible that the combination of extensive material and an extended timeframe contributed to some students losing track, especially those unfamiliar with the flipped model [11]. Given mixed evidence on the most effective media for content delivery [12], a cautious approach would be to test whether more tightly scaffolded, tailored learning activities with clear progression guidelines may improve engagement compared with offering an open choice between videos and readings.

Although we included quizzes, reflection tasks and assignments aimed at promoting active learning, students asked for additional learning activities that would let them apply knowledge. Given adult learners’ problem-centered orientation [17], this request is not surprising. However, because pre-class activities and teaching sessions were not mandatory, and students faced concurrent demands from work and private life, we may not have fully anticipated this preference. In line with Mishall et al.’s [36] recent recommendations, our findings indicate it would be useful to investigate whether adding active pre-class activities actually improves depth of understanding and critical thinking.

Some students reported that in-class teaching repeated pre-class content, while others found the approach beneficial. For students not to get frustrated and lose value of pre-class learning, it is essential that the in-class activities align with the pre-class work and that the pre-class work is not repeated in class [12]. We typically began in-class sessions with a brief review to establish common understanding, and it is possible that students felt this review was too similar to their pre-class preparation. Our analysis also found that students recalled prior large-group experiences in which the faculty assigned pre-class work but then covered the same content in class without holding students accountable, a pattern noted in earlier research [12]. This historical tendency could partly explain low preparation rates, though other factors (e.g., workload, assessment, or student expectations) may also contribute and warrant further study.

Throughout this project we became increasingly aware that accommodating pre-class preparation for large-group teaching may require a collective faculty effort. We recognize that the flipped approach is best considered a shared pedagogical responsibility. Accordingly, we have informed colleagues and sought consensus on common expectations for pre-class preparation and in-class activities. These efforts were intended to reduce students’ workload and conflicting demands and might help foster a culture of active learning in which pre-class preparation is expected for both large- and small-group teaching when appropriate. Further evaluation is needed to determine whether these measures may produce the intended effects.

Most students in our study prepared for small-group teaching and expressed a preference for it over large-group formats. They valued a safe environment with an available teacher to facilitate questions. Although we provided an online discussion forum for instant questions and help during the out-of-class activities, as recommended in previous research [11], relatively few students used it. In an era of expanding technological possibilities and tightening budgets, the flipped approach is sometimes proposed as a way to deliver more cost-effective, student-centered curricula [3]. However, van Alten et al. [6] reported higher learning outcomes in flipped classrooms when face-to-face time was not reduced compared with non-flipped classrooms. This suggests that multiple decisions should be considered and evaluated within each specific context, and further research is needed to clarify which format best supports learning.

We aimed to improve our teaching practice by testing and reflecting upon an action, but curricular changes and resource constraints prevented continuation as originally planned. Nevertheless, we have used our findings to inform further development of teaching and to encourage a shared culture around pre-class preparation. Faculty members were invited to reconsider pedagogical approaches, and we have observed a possible increase in the use of student-active teaching, particularly in small groups, although this has not been systematically evaluated. For large-group teaching, further exploration of the flipped classroom and other student-centered methods is warranted.

### Limitations

The study is limited by a small sample size and a low response rate, which may introduce non-response bias and limit generalizability. We have no further information about responders’ preparation for class beyond the information we assessed at this one point of time. With the small sample, we cannot rule out the possibility that it was the most motivated, engaged or satisfied students who participated, and that they may differ systematically from non-responders. Because the sample came from a single course at one educational institution, generalizability is further limited.

The study must also be interpreted in light of the institutional framework for mandatory teaching at the time of data collection [39]. Mandatory activities were required to have an academic justification anchored in the course’s learning outcomes and to involve assessable active student participation (e.g., skills training, clinical placements, simulation, group discussions, and seminars with submission of group work). Large-group sessions could be made mandatory if they addressed topics, methods, or knowledge that students could not reasonably acquire in other ways. In the teaching of EBP, only the post-class written assignment was mandatory. Attendance at the large-group sessions was voluntary because the knowledge was deemed attainable through self-study. This context likely contributed to the attendance rates observed in our cohorts and limits the transferability of our findings to settings or countries where physical attendance in teaching sessions is more strictly required or regulated.

Survey measurements were developed for this study and were not tested for measurement properties prior to use. Self-reported data may be affected by inaccurate self-assessment and social desirability bias. Findings should therefore be interpreted with caution. However, triangulation in data gathering, analysis and interpretation increases trustworthiness [27].

We did not access learning analytics from the learning management system (Canvas), such as log-ins, time spent on materials, or completion of pre-class activities. Consequently, we were not able to quantitatively examine students’ actual engagement with the pre-class materials or determine whether non-attending students had nonetheless engaged with the online materials. In retrospect, such data could have provided a useful indication of patterns and levels of engagement with the pre-class preparation.

The project group included faculty members with different levels of teaching experience. Although we intentionally separated teaching and evaluation roles to minimize bias, the project group’s pedagogical perspectives may nevertheless have influenced the design of the action and the interpretation of findings. We sought to mitigate this risk through a reflexive analytic approach.

Finally, we considered including students in the project group, but did not do so for pragmatic reasons (time constraints and organizational factors). Involving student representatives at all stages could have enriched this action research and is recommended for future iterations.

## 5. Conclusions

Using a systematic, iterative SoTL-inspired action research approach, we tested and refined strategies to support pre-class preparation for large-group EBP teaching across two cohorts. Through reflection on practice, implementation of a targeted action, and evaluation of outcomes, we gained practical insights into making pre-class work more feasible and meaningful for nursing students. Motivation emerged as central to pre-class preparation, and faculties can support it through clear communication of relevance, a well-organized learning platform, activating pre-class activities, and timely reminders. Successfully accommodating pre-class preparation for large-group teaching also appears to require coordinated faculty engagement and a shared commitment to student-centered approaches. Further evaluation is needed to determine which specific configurations work best in different contexts.

## Figures and Tables

**Table 1:** Thematical analysis with theme, sub-themes and excerpts.

Theme	Sub-Themes	Excerpts
Students need motivation for pre-class preparation	Information: Communicate relevance and provide timely reminders	*“I don’t really understand why it’s important to prepare, and I often don’t see the value until I’m in the lecture and understand the topic.” (Interview 2.3)* *“That it is communicated by the lecturer in advance of the lecture,* e.g., *at the end of the lecture a couple of days before and as an announcement in Canvas.” (Survey 2)*
	Organization: Learning platform and workload	*“It was very clear where to go to find the different things. It wasn’t just a case of being told to do something and then sitting there not knowing where to find it.” (Interview 1.1)* *“I found the information a bit overwhelming, especially when you suddenly had to do so much before a lecture.” (Interview 2.4)* *Pick out the key points. Say which things people should do, list the top priorities first and then the others. That way at least the most important stuff is noticed.” (Interview 2.3)*
	Engage learners: Diverse, interactive and aligned learning activities	*“… you learn by using all senses. You can have some material on video, read something, and do exercises.” (Interview 2.5)* *“… you have to search a bit and use your head a bit in that way; it’s often the case that if you just read, it doesn’t sink in.” (Interview 2.2)* *“If you’ve prepared for large-group lectures … you don’t get to use it or talk about it, but in a small group you get to discuss and talk things over with each other.” (Interview 2.2)* *“We have received preparation before a class, but I have often been left with the feeling afterwards that this was a bit doubled up.” (Interview 2.1)*

**Table 2:** Characteristics of participants for cohort 1 and cohort 2.

Results	Cohort 1 (*n* = 27) *n* (%)	Cohort 2 (*n* = 54) *n* (%)	*p*-Value
Gender			0.72 *
Female	23 (85)	48 (89)	
Male	4 (15)	6 (11)	
Prior studies at university/university college			0.54 *
Yes	6 (22)	17 (31)	
No	21 (78)	37 (69)	
Age			0.88 **
*N*	26	38	
Mean (SD)	22.7 (4.3)	22.3 (3.1)	
Min–Max	19–38	19–34	

* Analyzed by Fisher’s Exact Test. ** Analyzed by Mann–Whitney U Test.

**Table 3:** Preparedness for classroom teaching for cohort 1 and cohort 2.

Results	Cohort 1 (*n* = 27) *n* (%)	Cohort 2 (*n* = 54) *n* (%)	*p*-Value
Usually prepared for large-group teaching			1.0 *
Yes	4 (15)	9 (17)	
Sometimes	20 (74)	42 (78)	
No	2 (7)	3 (6)	
Usually prepared for small-group teaching			0.38 *
Yes	15 (56)	39 (72)	
Sometimes	9 (33)	13 (24)	
No	2 (7)	2 (4)	
Prepared for the day’s teaching session			0.18 **
Yes	16 (62)	41 (76)	
No	10 (37)	13 (24)	
Preparation for the day’s teaching session			
Resources on Canvas	12	35	
Read textbook	3	11	
Read, not specified	2	3	
Reasons for lack of preparation			
Lack of time	4	6	
Do not have textbook	3	0	
Not motivated	2	4	
Sick	1	1	
Other: tired, did not find info, not mandatory	0	4	

* Analyzed by Fisher–Freeman–Halton Exact Test. ** Analyzed by Fisher’s Exact Test.

## Data Availability

The datasets generated and analyzed during the current study are available from the corresponding author upon reasonable request by qualified researchers due to privacy or ethical restrictions.

## Public Involvement Statement

No public involvement in any aspect of this research.

## Guidelines and Standards Statement

The mauscript was drafted in line with Best practices in the reporting of participatory action research [23].

## References

[1] Frenk J., Chen L., Bhutta Z.A., Cohen J., Crisp N., Evans T., Fineberg H., Garcia P., Ke Y., Kelley P. (2010). Health professionals for a new century: Transforming education to strengthen health systems in an interdependent world. Lancet.

[2] Betihavas V., Bridgman H., Kornhaber R., Cross M. (2016). The evidence for ‘flipping out’: A systematic review of the flipped classroom in nursing education. Nurse Educ. Today.

[3] O’Flaherty J., Phillips C. (2015). The use of flipped classrooms in higher education: A scoping review. Internet High. Educ..

[4] Naing C., Whittaker M.A., Aung H.H., Chellappan D.K., Riegelman A. (2023). The effects of flipped classrooms to improve learning outcomes in undergraduate health professional education: A systematic review. Campbell Syst. Rev..

[5] Banks L., Kay R. (2022). Exploring flipped classrooms in undergraduate nursing and health science: A systematic review. Nurse Educ. Pract..

[6] van Alten D.C.D., Phielix C., Janssen J., Kester L. (2019). Effects of flipping the classroom on learning outcomes and satisfaction: A meta-analysis. Educ. Res. Rev..

[7] Baig M.I., Yadegaridehkordi E. (2023). Flipped classroom in higher education: A systematic literature review and research challenges. Int. J. Educ. Technol. High. Educ..

[8] Khazaei M.R., Moradi E., Barry A., Keshavarzi M.H., Hashemi A., Ramezani G., Zazoli A.Z., Farzadnia F. (2025). Effect of flipped classroom method on the reflection ability in nursing students in the professional ethics course; Solomon four-group design. BMC Med. Educ..

[9] Mitsiou D. (2019). The flipped classroom learning model as a means for acquiring the 21st century skills. J. Contemp. Educ. Theory Res..

[10] Strelan P., Osborn A., Palmer E. (2020). The flipped classroom: A meta-analysis of effects on student performance across disciplines and education levels. Educ. Res. Rev..

[11] Akçayır G., Akçayır M. (2018). The flipped classroom: A review of its advantages and challenges. Comput. Educ..

[12] Larson M.P., Linnell J. (2023). Are Students Coming to Class Prepared? The Importance of Pre-Class Learning in a Flipped Classroom. Issues Account. Educ..

[13] Youhasan P., Chen Y., Lyndon M., Henning M.A. (2021). Exploring the pedagogical design features of the flipped classroom in undergraduate nursing education: A systematic review. BMC Nurs..

[14] Al-Zahrani A.M. (2015). From passive to active: The impact of the flipped classroom through social learning platforms on higher education students’ creative thinking. Br. J. Educ. Technol..

[15] Zainuddin Z., Attaran M. (2016). Malaysian students’ perceptions of flipped classroom: A case study. Innov. Educ. Teach. Int..

[16] Lin H.-C., Hwang G.-J. (2019). Research trends of flipped classroom studies for medical courses: A review of journal publications from 2008 to 2017 based on the technology-enhanced learning model. Interact. Learn. Environ..

[17] Knowles M.S. (1990). The Adult Learner: A Neglected Species.

[18] Kunnskapsdepartementet (2017). Quality Culture in Higher Education [Kultur for Kvalitet I Høyere Utdanning].

[19] Norwegian Directorate for Higher Education and Skills Status Report on Higher Education [Tilstandsrapport for Høyere Utdanning 2025] 2025. https://hkdir.no/rapporter-undersokelser-og-statistikk/les-rapporten/tilstandsrapport-for-hoyere-utdanning-2025/finansiering-av-uh-sektoren.

[20] Ng E.K.L. (2023). Student engagement in flipped classroom in nursing education: An integrative review. Nurse Educ. Pract..

[21] Liu Y.-Q., Li Y.-F., Lei M.-J., Liu P.-X., Theobald J., Meng L.-N., Liu T.-T., Zhang C.-M., Jin C.-D. (2018). Effectiveness of the flipped classroom on the development of self-directed learning in nursing education: A meta-analysis. Front. Nurs..

[22] Barranquero-Herbosa M., Abajas-Bustillo R., Ortego-Maté C. (2022). Effectiveness of flipped classroom in nursing education: A systematic review of systematic and integrative reviews. Int. J. Nurs. Stud..

[23] Özbay Ö., Çınar S. (2021). Effectiveness of flipped classroom teaching models in nursing education: A systematic review. Nurse Educ. Today.

[24] Tan C., Yue W.-G., Fu Y. (2017). Effectiveness of flipped classrooms in nursing education: Systematic review and meta-analysis. Chin. Nurs. Res..

[25] Torres-Cano V., Vallbona-González A.M., Mondejar-Pont M. (2025). Nursing students’ outcomes in the flipped classroom approach: An integrative review. Teach. Learn. Nurs..

[26] Miller-Young J., Yeo M. (2015). Conceptualizing and Communicating SoTL: A Framework for the Field. Teach. Learn. Inq. ISSOTL J..

[27] McNiff J. (2017). Action Research: All you Need to Know.

[28] Smith L., Rosenzweig L., Schmidt M. (2010). Best Practices in the Reporting of Participatory Action Research: Embracing Both the Forest and the Trees. Couns. Psychol..

[29] Western Norway University of Applied Sciences Learning Management System: Canvas 2025. https://www.hvl.no/en/hvl-students/learning-management-system/.

[30] Western Norway University of Applied Sciences (2021). EBP Online [KBP på Tvers]: Western Norway University of Applied Sciences. https://www.hvl.no/om/organisering/fhs/kbp-pa-tvers/.

[31] Nortvedt M.W., Graverholt B., Jamtvedt G., Gundersen M.W. (2021). Work Evidencebased!—A Workbook [Jobb Kunnskapsbasert!: En Arbeidsbok].

[32] Dahlgren A. (2026). Behind the Headline [Bak Overskriftene].

[33] TimeEdit Acadamia Runs on TimeEdit 2025. https://www.timeedit.com/.

[34] IBM (2025). IBM Support.

[35] Braun V., Clarke V. (2006). Using thematic analysis in psychology. Qual. Res. Psychol..

[36] Mishall P.L., Meguid E.M.A., Elkhider I.A., Khalil M.K. (2025). The Application of Flipped Classroom Strategies in Medical Education: A Review and Recommendations. Med. Sci. Educ..

[37] Fontaine G., Vinette B., Weight C., Maheu-Cadotte M.-A., Lavallée A., Deschênes M.-F., Lapierre A., Castiglione S.A., Chicoine G., Rouleau G. (2024). Effects of implementation strategies on nursing practice and patient outcomes: A comprehensive systematic review and meta-analysis. Implement. Sci. IS.

[38] Ritter N., Arslan-Ari I. (2023). The Flipped Classroom Approach in High School Psychology: An Action Research Study. TechTrends.

[39] Department of Educational Quality (2020). Guidelines with Criteria for the Use of Compulsory Learning Activities–Adopted by the Education Committee 13.11.2019 [Rettleiar med Kriterium for Bruk av Obligatoriske Læringsaktivitetar-Vedtatt av Utdanningsutvalet 13 November 2019].

